# From Proteostasis Disruption to Stone Formation: Ubiquitin-Dependent Regulatory Networks in Calcium Oxalate Nephrolithiasis

**DOI:** 10.3390/biomedicines14102316

**Published:** 2026-10-12

**Authors:** Guanwei Wu, Hualin Liu, Weiyu Zhu, Yingqing Liu, Haibo Qin, Lingsong Tao

**Affiliations:** 1 Department of Urology, Affiliated Wuhu Hospital of East China Normal University, Wuhu 241001, China; wuguanwei1989@163.com (G.W.);; 2 Graduate School, Wannan Medical University, Wuhu 241001, China; 3 Department of Ultrasound, Affiliated Wuhu Hospital of East China Normal University, Wuhu 241001, China

**Keywords:** calcium oxalate nephrolithiasis, ubiquitin–proteasome system, deubiquitinating enzymes, proteostasis, crystal adhesion, regulated cell death

## Abstract

Calcium oxalate (CaOx) nephrolithiasis is a multistep disease process in which urinary supersaturation and crystallization interact with renal tissue responses to promote persistent mineral deposition and stone development. Two related pathological settings are particularly relevant: tubular crystal–epithelial interactions, where crystal-induced cellular stress, inflammation, regulated cell death, and epithelial surface remodeling favor crystal adhesion and retention; and Randall’s plaque (RP)-associated papillary mineralization, where interstitial mineral deposition and plaque exposure provide a substrate for CaOx overgrowth and stone initiation. Although pathologically distinct, both involve changes in protein homeostasis and cellular phenotype. The ubiquitin–proteasome system (UPS) and deubiquitinating enzymes (DUBs), through reversible control of protein stability, localization, and signaling, may regulate multiple steps in these processes. This review examines ubiquitin-dependent regulation across tubular injury and crystal retention, inflammatory signaling, cellular stress and organelle homeostasis, regulated cell death, and RP-associated remodeling. Disease-specific studies have identified mechanistically resolved pathways, including the VHL–BICD2–STAT1, Lgals3–TRIM21–PKM2, and PRMT1–UBE2M–NEDD4–PPARγ axes in CaOx-associated tubular pathology, as well as the OMD–NEDD4–BMP2 and TRAF1–TRAF2/CBLC axes in RP-associated pathology. Other findings implicate ubiquitin-dependent regulation in mitochondrial quality control, inflammation, epithelial remodeling, and cell death, but the evidence remains uneven. Only a limited number of E3 ligase/DUB–substrate relationships have been established directly in CaOx or RP models, while many candidate mechanisms derive from other renal or non-renal disease contexts. Viewed through the lens of proteostasis, altered protein fate may connect cellular stress with epithelial dysfunction, crystal retention, and papillary mineralization without implying a single route to stone formation. Defining CaOx-specific ubiquitin networks will require quantitative ubiquitinomics and ubiquitin-chain analysis integrated with spatial approaches and causal validation in disease-relevant models and human tissues. Such studies may reveal therapeutically tractable targets with sufficient molecular and renal selectivity.

## 1. Introduction

Kidney stone disease is a common and recurrent urological disorder that imposes a substantial health burden worldwide [1,2]. Its prevalence varies across geographic regions and populations, and the burden of urolithiasis has increased in many parts of the world over recent decades [1,2,3]. Recurrence remains a major clinical challenge, with a considerable proportion of patients experiencing additional stone episodes after an initial event [4,5]. Calcium-containing stones account for approximately 80% of urinary calculi, with calcium oxalate (CaOx) representing the predominant mineral component [6]. Although urinary supersaturation, nucleation, crystal growth, and aggregation provide the physicochemical basis for CaOx stone formation, crystal persistence and stone development also depend on biological processes within the kidney. Tubular epithelial injury and subsequent crystal adhesion and retention are increasingly recognized as important components of this process [6,7,8,9,10].

Renal tubular epithelial cells rely on tightly regulated protein-folding and degradative pathways to maintain cellular homeostasis under stress [11]. Oxalate and CaOx crystal exposure can disrupt this balance, inducing oxidative and inflammatory injury, endoplasmic reticulum (ER) stress, and other disturbances associated with impaired protein homeostasis [8,9,10,11,12]. Proteostasis disruption may therefore provide a mechanistic interface through which sustained crystal-induced stress alters tubular epithelial function and promotes a crystal-retentive environment.

The ubiquitin–proteasome system (UPS) is a principal pathway governing selective protein degradation and proteostasis, whereas deubiquitinating enzymes (DUBs) remove or remodel ubiquitin modifications to regulate substrate stability and signaling [13,14]. Ubiquitin-dependent regulation is integrated with oxidative stress, inflammation, regulated cell death, and tissue remodeling and has been implicated in diverse forms of kidney injury [14,15]. Importantly, direct CaOx studies have begun to identify specific ubiquitin-regulatory mechanisms linking altered protein fate to tubular injury and crystal deposition [16]. Nevertheless, disease-specific evidence remains limited, and the relevant E3 ligases, DUBs, substrates, ubiquitin-chain architectures, and cell-specific regulatory circuits remain incompletely defined.

This review critically examines ubiquitin-dependent regulation in CaOx nephrolithiasis and Randall’s plaque across crystal adhesion and retention, cellular stress and proteostasis, inflammation, mitochondrial quality control, autophagy, and regulated cell death. Direct evidence from CaOx- and Randall’s-plaque-associated models is distinguished from mechanistic evidence derived from other renal or non-renal systems. Within this evidence-based framework, we assess how ubiquitin-dependent regulation may connect crystal-induced cellular stress with persistent crystal retention and plaque-associated stone development, and discuss the translational implications and experimental priorities required to define CaOx-specific ubiquitin-regulatory networks.

## 2. Literature Search Strategy

A structured literature search was conducted in PubMed/MEDLINE, Web of Science Core Collection, and Scopus from database inception through 24 September 2026, to identify studies relevant to ubiquitin-dependent regulation in calcium oxalate (CaOx) nephrolithiasis and Randall’s plaque (RP). Search terms combined CaOx/stone-related concepts with terms related to ubiquitination, the ubiquitin–proteasome system (UPS), E3 ubiquitin ligases, deubiquitination, proteasomal degradation, protein stability, and ubiquitin-like modifications. No restrictions were applied for language, publication year, or document type. The complete database-specific search strategies and screening process are provided in Supplementary Table S1 and Supplementary Figure S1. Two authors independently screened the retrieved records for relevance to CaOx/RP-specific ubiquitin-dependent regulation, and disagreements regarding eligibility or evidence classification were resolved by discussion and consensus.

Original mechanistic studies directly investigating CaOx nephrolithiasis and RP were prioritized. Given the limited disease-specific evidence, targeted supplementary searches were also performed to identify mechanistically informative studies on ubiquitin-dependent regulation of CaOx-relevant pathological processes. Studies from renal disease models were prioritized where available, while selected non-renal studies were considered when they provided relevant mechanistic evidence. Disease-specific studies were classified as Level I (direct CaOx/RP-specific evidence for ubiquitin-dependent regulation) or Level II (supportive CaOx-context evidence without a complete disease-specific ubiquitin causal chain), whereas studies derived from other renal or non-renal systems were treated as contextual mechanistic evidence. The corresponding targeted search strategies are provided in Supplementary Table S1.

## 3. The Ubiquitin–Proteasome System and Deubiquitination: A Mechanistic Overview

### 3.1. Ubiquitination and Proteasomal Degradation

Ubiquitin is a highly conserved 76-amino-acid protein that serves as a reversible post-translational modifier. Its covalent attachment to substrate proteins can regulate protein stability, localization, molecular interactions, and activity, thereby influencing proteasomal degradation as well as diverse cellular signaling processes [13,17,18]. Ubiquitination should be distinguished from ubiquitin-like modifications such as neddylation, which involves covalent conjugation of NEDD8 rather than ubiquitin, although these modifications may interact within regulatory cascades.

Ubiquitin conjugation proceeds through a sequential enzymatic cascade involving ubiquitin-activating enzymes (E1s), ubiquitin-conjugating enzymes (E2s), and ubiquitin ligases (E3s), with E3 ligases providing much of the substrate specificity within the system [17,19]. Repeated ubiquitin transfer can generate distinct ubiquitin-chain topologies with different functional consequences. K48-linked polyubiquitin is predominantly associated with proteasomal degradation, whereas K63-linked chains more commonly participate in non-proteolytic signaling; other linkage types further expand the functional diversity of the ubiquitin code [20,21]. Thus, the biological outcome of ubiquitination depends on the modified substrate, chain topology, modification site, and cellular context.

Proteins marked for degradation are primarily processed by the 26S proteasome, which recognizes, unfolds, and proteolytically degrades ubiquitinated substrates [22,23]. Together with the autophagy–lysosomal system, the UPS contributes to intracellular proteostasis by controlling the turnover of regulatory, damaged, and misfolded proteins and coordinating protein quality control under cellular stress [24,25,26].

### 3.2. Deubiquitinating Enzymes: Reversibility and Specificity of Ubiquitin Signaling

Ubiquitin conjugation is dynamically counterbalanced by deubiquitinating enzymes (DUBs), which remove ubiquitin from substrates, trim or remodel ubiquitin chains, and recycle ubiquitin. Human DUBs are commonly classified into seven major families: ubiquitin-specific proteases (USPs), ubiquitin C-terminal hydrolases (UCHs), ovarian tumor proteases (OTUs), Machado–Joseph disease/Josephin proteases, MINDY family proteases, ZUFSP/ZUP1, and JAB1/MPN/MOV34 metalloenzymes (JAMMs), with USPs representing the largest group [27,28].

By removing degradation-associated ubiquitin chains or remodeling signaling-associated ubiquitin modifications, DUBs can alter substrate stability and the magnitude, duration, or specificity of cellular signaling [27,28,29,30,31]. Ubiquitin-dependent regulation therefore reflects a dynamic balance between conjugation and deconjugation rather than a unidirectional degradation pathway. Disruption of this balance can alter the abundance or activity of stress-responsive proteins and perturb cellular proteostasis [14,29]. Although DUBs have consequently attracted interest as pharmacological targets, achieving substrate and context specificity remains challenging [27,32].

**Figure 1:**
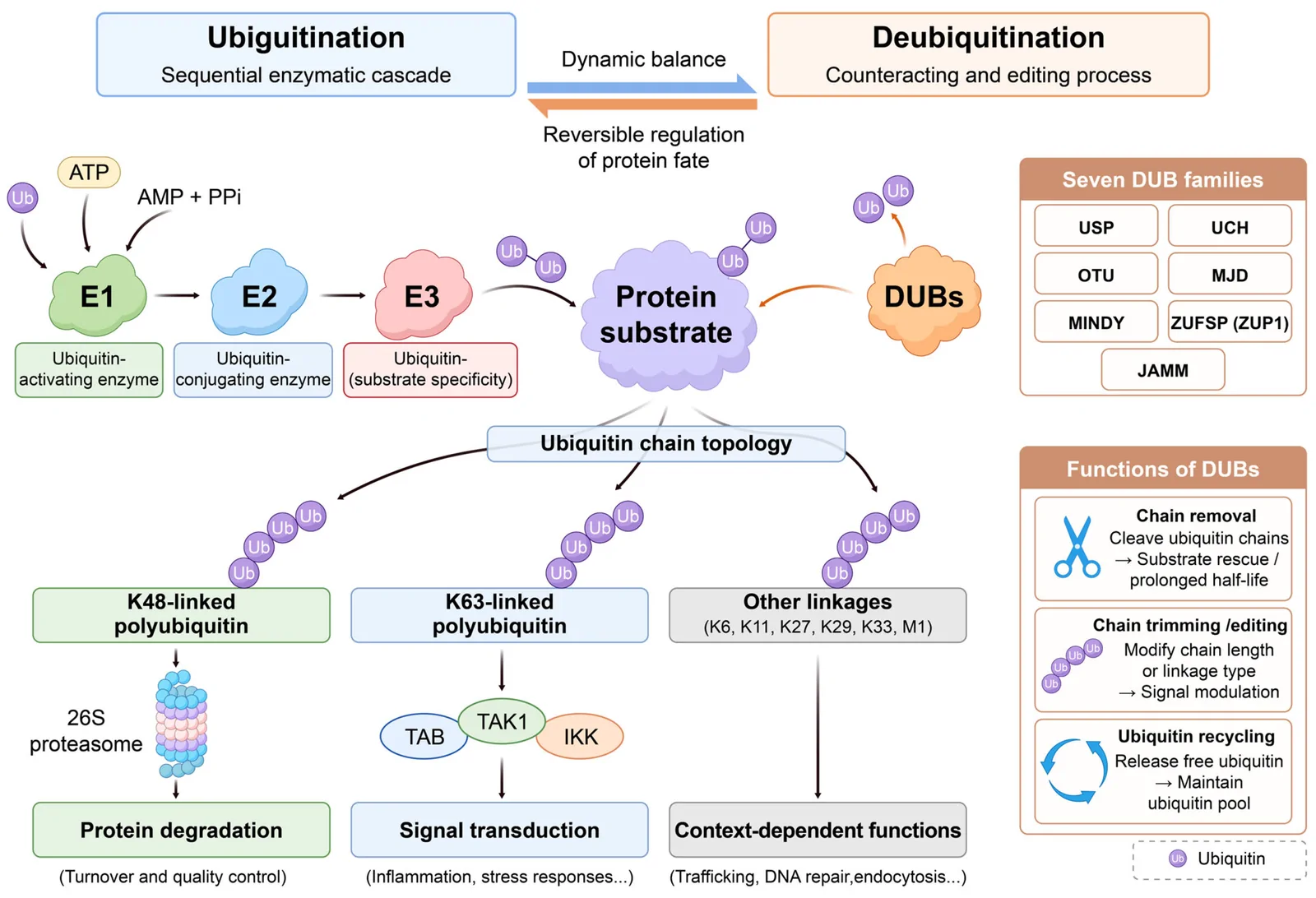
Overview of ubiquitination, deubiquitination, and ubiquitin-dependent protein fate.

In CaOx nephrolithiasis, this regulatory framework raises the disease-specific question of whether altered ubiquitin conjugation or deubiquitination modifies proteins governing tubular stress responses, crystal-interacting phenotypes, inflammation, and regulated cell death. The principal mechanisms of ubiquitination, deubiquitination, and ubiquitin-dependent protein fate discussed in this review are summarized in Figure 1.

Ubiquitination is mediated by the sequential actions of E1 ubiquitin-activating enzymes, E2 ubiquitin-conjugating enzymes, and E3 ubiquitin ligases, resulting in covalent attachment of ubiquitin to substrate proteins. Distinct ubiquitin-chain architectures confer different functional outcomes: K48-linked polyubiquitination is primarily associated with proteasomal degradation, whereas K63-linked chains more commonly participate in non-proteolytic signaling, with other linkage types further expanding the functional diversity of ubiquitin signaling. Deubiquitinating enzymes (DUBs) counterbalance ubiquitination by removing or remodeling ubiquitin modifications, thereby regulating ubiquitin-dependent protein fate and signaling. Major DUB families and their principal functions are also summarized.

## 4. Cellular and Molecular Landscape of Calcium Oxalate Nephrolithiasis

CaOx nephrolithiasis reflects the interplay between physicochemical crystallization and biological processes within the kidney. Urinary supersaturation provides the thermodynamic basis for crystal nucleation and growth, whereas the persistence and renal retention of crystals are influenced by tubular epithelial injury, cellular stress, inflammatory activation, regulated cell death, and remodeling of the crystal-interacting epithelial surface [6,8,10]. These processes can reinforce one another, as injury-associated cellular responses promote crystal adhesion and retention while retained crystals perpetuate epithelial and inflammatory stress [33,34]. In parallel, papillary interstitial mineralization and the development of Randall’s plaque provide a distinct pathological substrate for the initiation and growth of many idiopathic CaOx stones. Together, tubular crystal–epithelial interactions and plaque-associated processes define complementary pathological contexts for understanding the cellular and molecular regulation of CaOx nephrolithiasis [35].

### 4.1. Tubular Epithelial Injury, Crystal Adhesion, and Retention

Urinary supersaturation with calcium and oxalate is a prerequisite for CaOx crystallization. Increased concentrations of lithogenic ions, together with reduced activity of crystallization inhibitors such as citrate, favor crystal nucleation, growth, and aggregation [6]. Yet, crystallization alone does not determine whether a clinically relevant stone will develop [36]. A critical step is whether newly formed crystals are cleared with urinary flow or retained within the kidney.

Tubular epithelial injury creates a biological interface that favors crystal retention. Sustained exposure to high oxalate concentrations or CaOx crystals can injure epithelial cells and alter cell-surface properties involved in crystal adhesion and handling [10,37,38]. Injured or regenerating tubular epithelial cells exhibit increased luminal expression of hyaluronic acid (HA), osteopontin (OPN), and CD44, generating a surface with greater affinity for CaOx crystals [8,39]. Inflammatory signaling can further reinforce this phenotype. Recent experimental evidence shows that IL-6/p38 MAPK signaling increases OPN and CD44 expression and promotes crystal–cell adhesion [40]. Once retained at the tubular surface, crystals are more likely to resist urinary clearance, undergo further growth and aggregation, and inflict additional epithelial damage. Crystal adhesion and epithelial injury can therefore form a reinforcing loop that converts transient crystallization into persistent intrarenal crystal deposition.

### 4.2. Cellular Stress and the Inflammatory Microenvironment

Oxalate and CaOx crystal exposure induces substantial oxidative stress in renal tubular epithelial cells. Excessive reactive oxygen species (ROS) can overwhelm endogenous antioxidant defenses, disrupt redox homeostasis, and contribute to organelle dysfunction and epithelial injury [9,41,42]. Consistently, interventions that reduce oxidative stress or reinforce antioxidant defenses attenuate tubular injury and intrarenal crystal deposition in experimental CaOx models [41,42].

Mitochondria serve as both targets and amplifiers of crystal-induced oxidative stress, and their dysfunction is closely integrated with ER stress and disturbed protein-folding homeostasis. CaOx exposure disrupts mitochondrial homeostasis and further promotes ROS accumulation, whereas restoration of protective mitochondrial pathways attenuates epithelial injury and crystal deposition [43,44,45]. ER stress is likewise activated during oxalate- or CaOx-induced tubular injury and can interact with mitochondrial stress to promote epithelial dysfunction and apoptosis [12,46]. In macrophages, CaOx crystals can additionally induce ER stress and lysosomal dysfunction, enhancing extracellular vesicle release and subsequent tubular injury and renal crystal deposition [47]. Thus, oxidative stress, mitochondrial dysfunction, ER stress, and disturbed protein-folding homeostasis form an interconnected cellular stress network during sustained crystal exposure.

Inflammatory activation can further amplify this injury environment. Macrophage polarization influences the inflammatory milieu surrounding injured tubular epithelium and subsequent crystal deposition [48], while CaOx exposure activates inflammatory pathways involving NF-κB [9], p38 MAPK [40], and the NLRP3 inflammasome [49,50,51]. NLRP3 provides a direct link between crystal sensing and inflammatory signaling, with CaOx crystals inducing NLRP3-dependent caspase-1 activation and IL-1β release [50]. Together, these inflammatory responses can reinforce cellular stress and epithelial injury, thereby favoring continued crystal retention and deposition.

### 4.3. Regulated Cell Death

Regulated cell death is an important consequence of sustained CaOx-induced stress and contributes to the loss of tubular epithelial integrity. In addition to apoptosis, experimental studies implicate ferroptosis, pyroptosis, and necroptosis in CaOx-associated renal injury and crystal deposition [52,53,54,55,56,57,58,59,60]. Although these pathways differ in their molecular execution, they converge on epithelial damage and can further amplify oxidative or inflammatory stress.

Ferroptosis has emerged as an increasingly recognized component of CaOx-associated tubular injury. Characterized by iron-dependent phospholipid peroxidation and impaired antioxidant defense, ferroptotic susceptibility is closely linked to glutathione peroxidase 4 (GPX4) activity. Experimental CaOx studies show that modulation of ferroptosis alters tubular injury and crystal deposition [44,55,56,57]. Recent evidence further indicates that arachidonic-acid-associated phospholipid remodeling and membrane peroxidative damage can enhance crystal–cell adhesion, directly linking ferroptosis-associated membrane injury to a crystal-retentive epithelial phenotype [58].

Pyroptosis, necroptosis, and apoptosis provide additional routes through which sustained crystal-induced stress can compromise tubular integrity. Activation of the NLRP3–caspase-1–GSDMD pathway has been demonstrated in experimental CaOx models, and attenuation of pyroptotic signaling reduces epithelial injury, crystal adhesion, or crystal deposition [33,53,54,59]. RIPK3-mediated necroptosis similarly contributes to tubular injury, inflammation, and intrarenal crystal deposition [52], whereas apoptosis remains a well-established response to oxalate and CaOx exposure and is closely associated with oxidative stress, mitochondrial dysfunction, and ER stress [41,46,60]. Collectively, these forms of regulated cell death integrate with oxidative stress, inflammatory signaling, and epithelial surface remodeling to reinforce the tubular injury–crystal retention cycle.

### 4.4. Randall’s Plaque and Papillary Mineralization

Beyond these tubular crystal–epithelial interactions, CaOx stone development is also closely associated with pathological processes occurring within the renal papilla, particularly the formation of Randall’s plaque (RP). RP consists predominantly of interstitial calcium phosphate deposits, mainly in the form of apatite, that originate in the basement membranes of thin loops of Henle and progressively extend into the surrounding papillary interstitium [61,62,63]. This interstitial pattern is pathologically distinct from intratubular crystal deposition associated with tubular injury and plugging [61,62]. In idiopathic CaOx stone formers, RP can extend toward the papillary surface and provide an important substrate for stone attachment and subsequent growth [64,65].

The mechanisms underlying RP development remain incompletely understood. Studies of human RP tissue indicate a complex papillary microenvironment involving interstitial mineralization, extracellular-matrix remodeling, and mineralization-associated cellular changes, although the mechanisms driving plaque initiation and expansion remain incompletely defined [66,67]. These observations support the involvement of biologically regulated processes in RP development.

As interstitial plaque expands toward the papillary surface, disruption of the overlying urothelium can expose the mineralized plaque to calyceal urine. Organic matrix and additional mineral phases can subsequently accumulate on the exposed plaque, followed by CaOx overgrowth and development of an attached stone [65]. RP therefore provides an important pathological substrate linking papillary interstitial mineralization to plaque-associated CaOx stone initiation. This pathway is related to, but pathologically distinct from, the tubular injury–crystal retention pathway described above.

## 5. Ubiquitin-Dependent Regulatory Networks in Calcium Oxalate Nephrolithiasis

The ubiquitin–proteasome system and deubiquitination participate in multiple regulatory processes relevant to CaOx nephrolithiasis by controlling protein stability, localization, and signaling. In the CaOx setting, ubiquitin-dependent regulation intersects with tubular epithelial injury, cellular stress and proteostasis disruption, inflammatory signaling, regulated cell death, and remodeling of the crystal-interacting epithelial surface [16,30,55,58,68,69]. Altered protein fate may consequently influence epithelial responses to oxalate and crystal exposure, contributing to persistent cellular dysfunction, crystal adhesion and retention, and renal crystal deposition.

**Figure 2:**
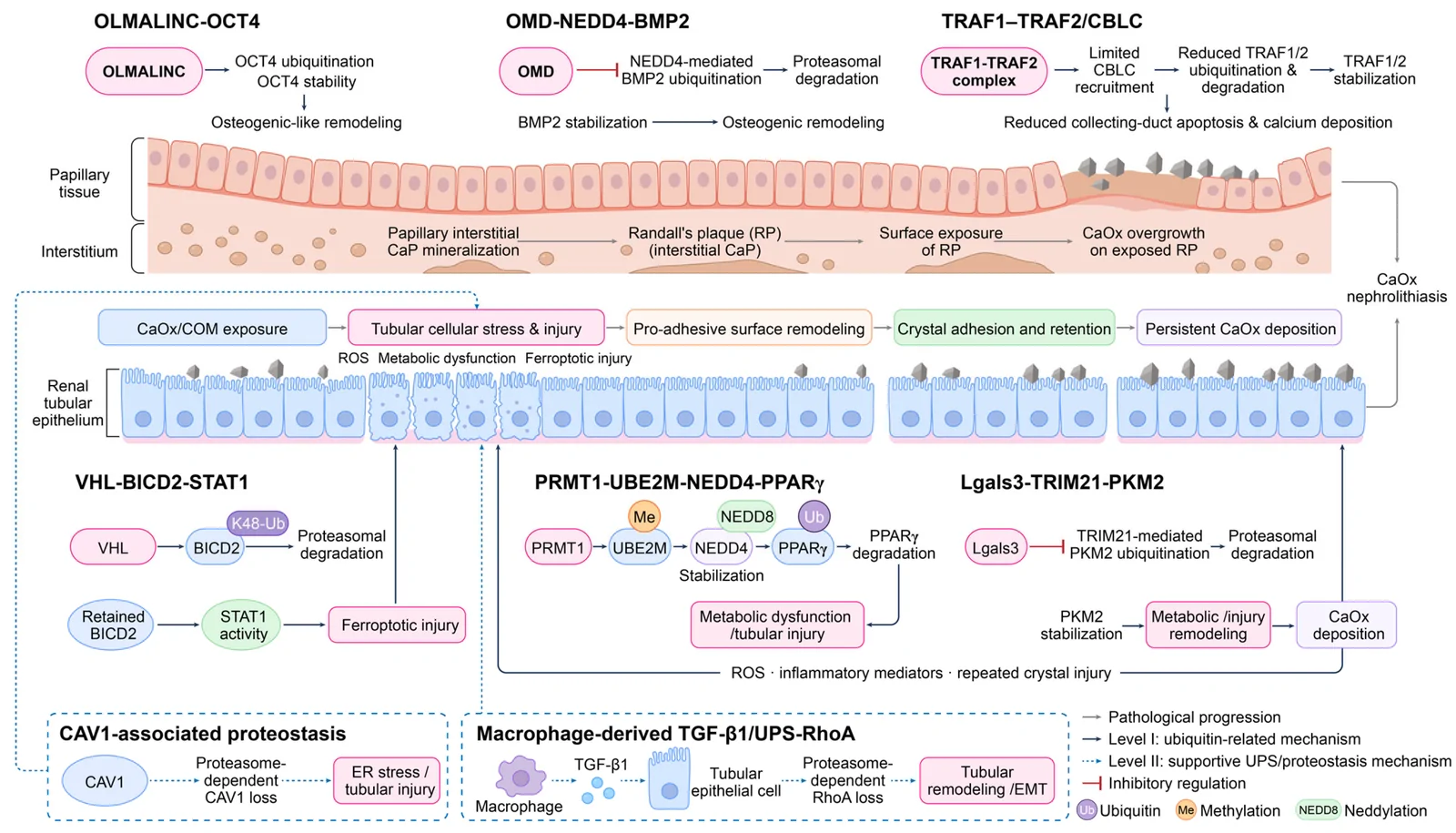
Integrated framework of ubiquitin-dependent regulatory networks in calcium oxalate nephrolithiasis.

The available evidence varies considerably in disease specificity and mechanistic resolution. Several ubiquitin-dependent mechanisms have been demonstrated directly in CaOx-associated experimental settings, while additional regulatory relationships have been characterized mainly in other renal or non-renal disease models and provide mechanistic context for CaOx pathogenesis. Recent studies of Randall’s plaque (RP) have further identified ubiquitin-related mechanisms associated with papillary mineralization and plaque development [70,71,72], extending the current evidence from tubular crystal–epithelial interactions to plaque-associated stone formation. Together, these findings outline a broader ubiquitin-dependent regulatory landscape encompassing tubular injury and crystal retention, cellular stress and proteostasis, inflammatory signaling, regulated cell death, and RP-associated papillary mineralization, as summarized in Figure 2.

Calcium oxalate (CaOx) nephrolithiasis is represented by two interconnected pathological routes: a tubular pathway involving CaOx/calcium oxalate monohydrate (COM)-induced cellular stress and injury, pro-adhesive surface remodeling, crystal adhesion and retention, and persistent CaOx deposition; and a papillary pathway involving interstitial calcium phosphate (CaP) mineralization, Randall’s plaque (RP) formation and surface exposure, and subsequent CaOx overgrowth. Representative CaOx/RP-specific ubiquitin-dependent mechanisms include the VHL–BICD2–STAT1, PRMT1–UBE2M–NEDD4–PPARγ, and Lgals3–TRIM21–PKM2 axes in tubular pathology, together with OLMALINC–OCT4, OMD–NEDD4–BMP2, and TRAF1–TRAF2/CBLC regulation in RP-associated pathology. CAV1-associated proteostasis and macrophage-derived TGF-β1/UPS–RhoA signaling represent supportive CaOx-context mechanisms in which proteasome-dependent regulation has been implicated but a complete substrate-specific ubiquitination mechanism has not been established. Gray solid arrows indicate relationships within the pathological framework. Within the ubiquitin-related regulatory modules, black solid lines indicate Level I direct CaOx/RP-specific evidence for ubiquitin-dependent regulation, whereas black dashed lines indicate Level II supportive CaOx-context evidence without a fully established substrate-specific ubiquitination mechanism.

### 5.1. Ubiquitin-Dependent Regulation of Tubular Injury, Crystal Adhesion, and Retention

Crystal adhesion and retention contribute to the persistence of CaOx crystals within the kidney. Following injury or phenotypic remodeling, tubular epithelial cells display increased availability of crystal-binding determinants such as hyaluronan (HA), osteopontin (OPN), and CD44 [8,39]. The functional importance of this surface remodeling is supported by experiments showing that OPN suppression reduces CaOx crystal binding to renal epithelial cells [73]. More recently, tubular epithelial cell-derived clusterin (CLU) was shown to promote COM adhesion and crystal growth; CLU knockdown reduced crystal binding to HK-2 cells, and tubular-specific Clu deletion decreased renal CaOx crystal deposition in vivo [74]. Yet the corresponding ubiquitin biology remains largely unresolved. No defined E3 ligase or DUB has been shown to directly regulate the principal crystal-binding determinants in response to CaOx exposure.

CaOx models have linked ubiquitin-dependent protein turnover to the tubular injury accompanying crystal retention. STUB1, for example, promotes CFTR ubiquitination and degradation and modulates ROS-associated autophagic responses in COM-treated HK-2 cells and glyoxylate-induced CaOx crystal deposition in mice [16]. A less resolved example involves CAV1. COM exposure reduces CAV1 protein abundance without a corresponding transcriptional decrease, whereas proteasome inhibition prevents this loss [75]. This finding implicates proteasome-dependent turnover but does not establish CAV1 as a directly ubiquitinated substrate or identify the responsible E3 ligase or DUB.

Potential connections to the crystal-binding machinery come mainly from other experimental systems. USP10 interacts with and deubiquitinates CD44 in breast cancer cells, increasing CD44 stability [76]. HAS2, the enzyme responsible for HA synthesis, is also regulated by DUBs: USP17 removes K48- and K63-linked polyubiquitin chains from HAS2 and stabilizes the protein, whereas USP4 preferentially reduces HAS2 monoubiquitination and has distinct effects on HA production [77]. These observations provide mechanistic precedents for ubiquitin-dependent control of two established components of the crystal-adhesive surface. Whether either regulatory relationship operates in CaOx-exposed renal tubular epithelium awaits direct investigation.

CaOx-specific studies increasingly define the proteins and epithelial changes associated with crystal adhesion, whereas substrate-specific ubiquitin regulation of this surface remains poorly characterized. Establishing E3/DUB–substrate relationships for CD44, HAS2/HA, OPN, or other crystal-interacting proteins—and testing their effects on crystal retention in vivo—represents an important next step toward defining a CaOx-specific ubiquitin network.

### 5.2. Ubiquitin-Dependent Regulation of Inflammatory Signaling

Inflammatory signaling accompanies tubular injury during sustained oxalate and CaOx crystal exposure and can reinforce a microenvironment favorable to crystal retention [48,49,50]. Among the pathways implicated, NF-κB and the NLRP3 inflammasome have the strongest experimental support in CaOx models [9,49,50]. Ubiquitin signaling is integral to both pathways, yet the E3 ligases and DUBs responsible for their regulation in the CaOx kidney remain poorly characterized.

One CaOx-context study linked proteasome-dependent turnover to macrophage–epithelial communication. CaOx-induced macrophage-derived TGF-β1 was associated with reduced epithelial RhoA, whereas proteasome inhibition restored RhoA abundance and attenuated epithelial–mesenchymal transition [78]. These results implicate proteasome-dependent protein turnover in inflammatory cell–epithelial communication during CaOx exposure, without establishing direct ubiquitination of RhoA. For NF-κB itself, the relevant ubiquitin machinery has been characterized largely outside stone models. IκBα degradation depends on K48-linked polyubiquitination, while non-degradative ubiquitin signaling at upstream complexes contributes to IKK activation [79]. A20 and CYLD can restrain this signaling through distinct ubiquitin-editing mechanisms involving receptor-proximal ubiquitin complexes and TRAF proteins [80,81]. Their roles during CaOx exposure remain unknown.

NLRP3 sits at a natural intersection between crystal-driven inflammation and ubiquitin control. CaOx crystals activate NLRP3, caspase-1, and IL-1β signaling in renal experimental systems [49,50], while studies in macrophage and other inflammatory models show that NLRP3 activity is strongly influenced by its ubiquitination state [82,83,84]. BRCC3 promotes inflammasome activation through NLRP3 deubiquitination [82], whereas TRIM31 drives K48-linked NLRP3 polyubiquitination and proteasomal degradation [83]. Other E3-ligase circuits add further complexity; RNF125-mediated K63 ubiquitination can facilitate Cbl-b recruitment and subsequent K48-linked ubiquitination of NLRP3 [84]. These studies establish the mechanistic capacity of ubiquitin editing to tune NLRP3 activity, but whether the same enzyme–substrate relationships operate in CaOx-exposed renal cells is unknown.

What is missing is disease-specific resolution at the enzyme–substrate level. NF-κB and NLRP3 are established components of CaOx-associated inflammation, but their upstream ubiquitin regulators have largely been inferred from other inflammatory systems. Whether CaOx exposure reshapes these E3/DUB–substrate relationships in specific renal cell populations, and whether such changes influence crystal retention in vivo, remains unresolved.

### 5.3. Ubiquitin-Dependent Regulation of Cellular Stress and Proteostasis

Sustained oxalate and CaOx crystal exposure disrupts protein and organelle homeostasis in renal tubular epithelial cells, alongside oxidative stress, ER stress, mitochondrial dysfunction, and metabolic disturbance [9,12,40,41,42,43,44,45]. The UPS and autophagy are closely integrated into these responses [15,25]. Recent studies provide direct evidence that altered ubiquitin-dependent protein turnover can influence the metabolic and stress responses of CaOx-exposed cells.

Lgals3 connects ubiquitin-dependent protein turnover to metabolic remodeling. Lgals3 is increased in CaOx-associated experimental models and human stone-related kidney tissue, and genetic manipulation of Lgals3 alters renal crystal deposition and injury [85]. Lgals3 interacts with PKM2 and interferes with TRIM21-mediated PKM2 ubiquitination and proteasomal degradation, thereby stabilizing PKM2 and sustaining glycolytic activity and lactate production. Increased H3K18 lactylation further connects altered PKM2 turnover with transcriptional responses associated with renal fibrosis [85]. A separate pathway links protein stability to fatty-acid metabolism. PRMT1-mediated methylation of UBE2M at R169 enhances NEDD4 neddylation and stability, allowing NEDD4 to promote PPARγ ubiquitination and degradation during CaOx-induced renal injury [86]. Here, methylation, neddylation, and ubiquitination act sequentially rather than interchangeably.

Other observations suggest that the disturbance extends beyond these pathways. Hyperoxaluria increases SIRT6 ubiquitination and its interaction with HSP70, followed predominantly by autophagy–lysosomal degradation [87]. Earlier experiments also reported reduced cellular ubiquitinated proteins together with accumulation of oxidized proteins after high-dose COM or COD exposure [88]. The latter finding does not identify individual substrates, but it suggests that the proteostatic disturbance accompanying crystal injury extends beyond the few substrate-specific pathways currently defined.

Protein quality control at the ER and mitochondria is less well resolved in stone disease. ER stress is consistently observed after oxalate and CaOx exposure [9,12,41,46], yet direct involvement of the SEL1L–HRD1 ERAD machinery or other ER-associated ubiquitin regulators has not been established [89,90,91,92]. Mitochondrial dysfunction and mitophagy, by contrast, have clear functional relevance to CaOx injury and crystal deposition [42,43,44,93,94]. PINK1–Parkin-mediated mitochondrial ubiquitination and its antagonism by USP30 provide a well-defined mechanism for selective mitochondrial quality control in other settings [95,96,97], but the extent to which this ubiquitin machinery is engaged during CaOx exposure remains unclear. This distinction matters when interpreting autophagy-related findings in CaOx models: autophagy induction, completion of autophagic flux, and selective mitophagy represent different biological processes and should not be treated as equivalent readouts [16,44,94,98,99,100,101].

Substrate-specific evidence is currently strongest for the Lgals3–TRIM21–PKM2 and PRMT1–UBE2M–NEDD4–PPARγ pathways. SIRT6 degradation and global changes in ubiquitinated proteins point to a wider disturbance of proteostasis, while ER- and mitochondria-associated ubiquitin networks remain less defined in CaOx disease. How these processes interact during persistent crystal exposure is still unresolved.

### 5.4. Ubiquitin-Dependent Regulation of Regulated Cell Death

Several forms of regulated cell death occur during oxalate and CaOx crystal injury, including ferroptosis, pyroptosis, apoptosis, and necroptosis [52,53,54,55,56,57,58,59,60]. Their ubiquitin-dependent regulation is unevenly resolved in stone disease. The strongest CaOx-specific evidence currently centers on ferroptosis, where altered protein turnover has been linked directly to ferroptotic susceptibility and crystal-associated renal injury.

One such mechanism operates upstream of the cystine/glutamate antiporter system Xc^−^. As the substrate-recognition component of a VHL-containing E3 ubiquitin ligase complex, VHL interacts with BICD2 in CaOx models and promotes its K48-linked polyubiquitination and proteasomal degradation. BICD2 facilitates STAT1 nuclear translocation and enhances IFNγ-mediated suppression of SLC3A2 and SLC7A11, thereby increasing susceptibility to lipid peroxidation and ferroptotic cell death. Manipulation of VHL or BICD2 alters the ferroptotic response to crystal exposure and the severity of crystal-associated renal injury in vivo [102]. The VHL–BICD2–STAT1 axis therefore represents a CaOx-specific ubiquitin-dependent mechanism linking K48-mediated protein turnover to ferroptosis.

GPX4 represents another ubiquitin-regulated node in ferroptosis susceptibility. In CaOx-associated models, Hsp90α interacts with GPX4 and promotes its ubiquitination and degradation, accompanied by increased lipid peroxidation, ferroptotic tubular injury, crystal adhesion, and renal CaOx deposition [55]. Hsp90α itself is not an E3 ubiquitin ligase, and the E3 responsible for GPX4 ubiquitination in this setting has not been identified. The ubiquitin linkage and modification site also remain unresolved. Evidence from other forms of renal injury shows that GPX4 turnover can be regulated by defined ubiquitin enzymes: TRIM21 promotes GPX4 ubiquitination and degradation during renal ischemia–reperfusion injury [103], whereas OTUD5 deubiquitinates and stabilizes GPX4 [104]. These mechanisms establish GPX4 as a substrate for bidirectional ubiquitin control in the kidney, but neither regulator has been connected to the Hsp90α-associated GPX4 destabilization observed during CaOx exposure.

The ubiquitin machinery underlying other forms of CaOx-associated cell death is much less defined. NLRP3-dependent pyroptotic signaling participates in crystal-induced renal injury [50,53,54,59], although its ubiquitin regulation has already been considered above in the context of inflammatory signaling. Direct evidence that NLRP3-directed E3 ligases or DUBs control pyroptosis during CaOx exposure is still lacking. Apoptosis is also consistently observed in experimental models of oxalate- and CaOx-crystal-induced injury, and inhibition of RIPK3-dependent necroptosis attenuates CaOx-associated renal injury [52,60]. CYLD, A20, and other ubiquitin regulators can modify RIPK signaling in experimental systems outside nephrolithiasis [105,106,107], but comparable E3/DUB–substrate relationships have not been established in CaOx models.

Ferroptosis remains the most clearly resolved form of ubiquitin-regulated cell death in CaOx nephrolithiasis. VHL–BICD2 provides a defined ubiquitin-dependent substrate-regulatory mechanism, whereas the Hsp90α–GPX4 pathway identifies disease-specific GPX4 ubiquitination without resolving the responsible E3 ligase. Whether ubiquitin-dependent regulation similarly shapes pyroptosis, apoptosis, or necroptosis during persistent crystal exposure remains an open question.

### 5.5. Ubiquitin-Dependent Regulation in Randall’s Plaque and Papillary Mineralization

Randall’s plaque (RP) provides a distinct pathological substrate for the initiation of many idiopathic CaOx stones. As discussed above, RP develops through calcium phosphate deposition within the papillary interstitium and can extend toward the papillary surface, where exposure to urine permits subsequent matrix accumulation and CaOx overgrowth. Ubiquitin-dependent protein stability has now been linked to two cellular processes underlying RP development: osteogenic-like remodeling of renal interstitial fibroblasts and apoptosis-associated calcium deposition around collecting ducts.

One such mechanism involves the lncRNA OLMALINC and OCT4. In human RP tissue, OCT4 is increased in association with osteogenic markers, while OLMALINC is enriched in RP-associated fibroblasts and positively correlated with OCT4 expression. In primary human renal interstitial fibroblasts, OLMALINC physically interacts with OCT4 and increases its protein stability without altering OCT4 mRNA abundance. OLMALINC knockdown shortens the half-life of OCT4, increases its ubiquitination, and reduces OCT4 protein levels—effects reversed by proteasome inhibition. Stabilized OCT4 promotes BMP2 transcription and osteogenic-like differentiation, linking altered OCT4 turnover to a cellular phenotype associated with RP formation [70]. The E3 ligase responsible for OCT4 ubiquitination in this pathway has not been identified.

The OMD–BMP2 pathway provides a defined E3–substrate relationship. OMD is enriched in RP tissue and promotes an osteogenic-like phenotype in human renal interstitial fibroblasts. OMD binds BMP2 and prolongs its protein stability by limiting NEDD4-mediated ubiquitination and proteasomal degradation. Silencing OMD increases the association between NEDD4 and BMP2 and enhances BMP2 ubiquitination, whereas NEDD4 knockdown or inhibition largely reverses these effects. The resulting stabilization of BMP2 reinforces BMP2/BMPR1A/RUNX2 signaling, with RUNX2 in turn promoting OMD transcription and establishing a positive feedback loop that favors an osteogenic-like interstitial microenvironment and calcium phosphate deposition [71].

Ubiquitin-dependent regulation also links collecting duct cell survival to early calcium deposition. M2 macrophage-derived exosomal TRAF2 enters AQP2^+^-collecting duct cells and forms a stable complex with intracellular TRAF1. CBLC was identified as a shared E3 ubiquitin ligase for both TRAF1 and TRAF2. Complex formation reduces CBLC recruitment, limiting ubiquitination and proteasomal degradation of both proteins. Stabilized TRAF1–TRAF2 signaling activates NF-κB1/NF-κB2-associated anti-apoptotic responses, reducing AQP2^+^ cell apoptosis and subsequent calcium deposition. Consistent with this mechanism, TRAF1 and TRAF2 were reduced in human RP tissue and associated with markers of apoptosis. TRAF1 suppression in AQP2^+^ cells was further confirmed in a tissue microarray containing paired RP and non-RP specimens. Manipulation of this pathway also altered calcium deposition in experimental RP-related models [72].

Together, OLMALINC–OCT4, OMD–NEDD4–BMP2, and TRAF1–TRAF2/CBLC illustrate three distinct modes of ubiquitin-related regulation within the plaque-forming microenvironment. These studies extend ubiquitin-dependent regulation in nephrolithiasis beyond tubular responses to CaOx crystals themselves, connecting altered protein stability to both osteogenic-like remodeling of the papillary interstitium and apoptosis-associated calcium deposition around collecting ducts.

Across these pathological processes, the evidence for ubiquitin-dependent regulation in CaOx nephrolithiasis varies considerably in disease specificity and mechanistic resolution. CaOx- and RP-specific studies have identified several substrate-level mechanisms involving protein ubiquitination, degradation, or stabilization, while other findings implicate proteasome-dependent turnover without establishing a defined E3/DUB–substrate relationship. These CaOx/RP-specific and supportive findings are summarized in Table 1. Additional ubiquitin-dependent mechanisms characterized in other renal or non-renal disease settings provide mechanistic context for processes relevant to CaOx nephrolithiasis, including epithelial surface remodeling, inflammatory signaling, and regulated cell death (Table 2). Collectively, these findings place ubiquitin-dependent protein fate at the intersection of tubular injury and crystal retention, cellular stress, inflammation, regulated cell death, and RP-associated papillary mineralization in CaOx nephrolithiasis.

## 6. Therapeutic Targeting of the Ubiquitin System in CaOx Nephrolithiasis

The mechanistic evidence discussed above identifies several ubiquitin-dependent pathways with potential therapeutic relevance to CaOx nephrolithiasis. Mechanistic validation of an E3 ligase, DUB, or ubiquitin-regulated substrate, however, does not by itself establish pharmacological tractability, and these pathways differ substantially in their proximity to therapeutic application. Systemic manipulation of ubiquitin-dependent protein regulation may disrupt essential proteostatic functions, while approaches designed to improve target or renal selectivity remain largely untested in CaOx disease. The therapeutic implications of these pathways therefore need to be considered in relation to the strength and disease proximity of the supporting evidence, ranging from mechanisms demonstrated directly in CaOx or RP models to observations derived from other disease settings and emerging technologies that could enable more selective intervention.

### 6.1. Therapeutic Relevance of CaOx-Validated Ubiquitin-Dependent Mechanisms

Several ubiquitin-dependent mechanisms identified in CaOx nephrolithiasis have been functionally validated in disease-relevant models. Suppression of STUB1 or restoration of CFTR attenuated tubular injury, and CFTR overexpression reduced renal injury and crystal deposition in vivo [16]. Disruption of the Lgals3–TRIM21–PKM2 pathway likewise reduced metabolic reprogramming, fibrosis, and CaOx deposition [85]. Together, these studies show that manipulating defined ubiquitin-regulatory relationships can modify CaOx-associated phenotypes.

Other CaOx studies implicate ubiquitin-dependent protein regulation in metabolic stress and ferroptosis. Tubular PRMT1 deficiency attenuated CaOx-associated injury by interrupting the UBE2M methylation–NEDD4 neddylation–PPARγ ubiquitination and degradation cascade [86]. In the ferroptotic response, VHL promotes K48-linked degradation of BICD2 [102], whereas Hsp90α-associated GPX4 ubiquitination accelerates GPX4 loss, although the responsible E3 ligase remains unknown [55]. Notably, Hsp90α inhibition or deficiency preserved GPX4 and reduced ferroptotic injury, supporting the therapeutic tractability of this pathway at the experimental level.

Ubiquitin-dependent regulation has also been implicated in papillary mineralization associated with RP. Manipulation of the OMD–NEDD4–BMP2 and TRAF1–TRAF2/CBLC axes altered mineralization-associated phenotypes in disease-specific models [71,72]. Yet, even for these CaOx- and RP-specific mechanisms, the available evidence largely establishes experimental tractability. Whether selective ubiquitin-directed intervention can prevent clinically relevant stone formation or recurrence is unknown, and selective pharmacological modulation, renal exposure, safety, and efficacy against stone outcomes remain to be established.

### 6.2. Therapeutic Insights from Ubiquitin Regulation in Other Disease Contexts

Mechanisms characterized outside CaOx nephrolithiasis offer additional clues to the tractability of pathological processes implicated in stone disease. In inflammatory signaling, A20 and related ubiquitin-editing regulators restrain NF-κB activation, while NLRP3 is regulated in opposing directions by the E3 ligase TRIM31 and the DUB BRCC3 [80,82,83]. Selective modulation of such nodes could, in principle, alter inflammatory signaling without globally suppressing the UPS. Whether these mechanisms operate similarly in CaOx nephrolithiasis remains uncertain, as none of these specific interventions has been validated in CaOx models.

Evidence from renal ferroptosis is more closely aligned with mechanisms already implicated in CaOx injury. In renal ischemic injury, TRIM21 promotes GPX4 ubiquitination and degradation, whereas OTUD5 stabilizes GPX4 and limits ferroptotic tubular injury [103,104], independently supporting GPX4 stability as a ubiquitin-sensitive node alongside the CaOx-specific Hsp90α–GPX4 pathway. More distant mechanistic parallels come from studies of epithelial surface proteins outside the kidney: USP10 stabilizes CD44, while USP17/USP4 regulate HAS2 [76,77]. Given the involvement of CD44 and HA in crystal–epithelial interactions, these observations raise testable questions about whether analogous ubiquitin-dependent regulation occurs in crystal-exposed renal tubular cells. At present, however, mechanisms derived from other renal or non-renal settings remain candidates for CaOx-specific testing rather than established therapeutic targets.

### 6.3. Emerging Strategies for Selective and Kidney-Targeted Intervention

Targeted protein degradation (TPD) offers a way to manipulate selected proteins through the ubiquitin–proteasome machinery without globally inhibiting the UPS. Proteolysis-targeting chimeras (PROTACs), for example, recruit an E3 ligase to a protein of interest, leading to its ubiquitination and proteasomal degradation [108]. A recently developed NLRP3-directed PROTAC recruited VHL and induced NLRP3 degradation, with a corresponding reduction in IL-1β release in cellular assays [109]. Although NLRP3 is relevant to CaOx-associated inflammation, this degrader has not been tested in nephrolithiasis models, and targeted degradation has yet to be applied to disease-validated proteins identified in CaOx or RP studies.

The spatial distribution of ubiquitin-regulatory pathways presents a different challenge. Receptor-mediated renal delivery offers one means of concentrating an intervention within selected nephron segments [110]. Megalin-targeted carriers, for instance, have increased drug accumulation in proximal tubular cells and improved renal injury in experimental ischemia–reperfusion models [111], and related polymeric systems have combined tubular targeting with intracellular or stimulus-responsive drug release [110]. Restricting exposure in this way could reduce systemic perturbation of proteostasis. In CaOx nephrolithiasis, however, kidney selectivity alone may be insufficient. Tubular crystal–epithelial injury and papillary/RP-associated mineralization occupy anatomically and biologically distinct compartments and may ultimately require different targeting strategies.

A further degree of selectivity could come from conditional degradation or drug release. Photocaged and ROS-responsive PROTAC systems have been developed to confine protein degradation to defined spatial or biochemical conditions [112,113]. In experimental kidney injury, ROS-responsive delivery systems have similarly coupled renal accumulation with preferential payload release under oxidative stress [114]. Whether such release can discriminate CaOx-associated oxidative microenvironments has not been examined. Oxidative stress in crystal-exposed tubular epithelium nevertheless provides a rationale for testing this approach, particularly if it can be coupled to an E3/DUB–substrate pathway already validated in CaOx disease. At present, TPD, kidney-targeted delivery, and conditional activation remain enabling technologies whose relevance to CaOx therapy will depend on disease-specific target selection, renal and cellular exposure, and preservation of physiological proteostasis.

### 6.4. Translational Barriers and Priorities for Clinical Development

Moving ubiquitin-directed strategies toward CaOx therapy will require more than identifying disease-associated targets. E3 ligases and DUBs often regulate multiple substrates and participate in essential physiological processes, so their effects may vary with the substrate, cell type, renal compartment, and stage of disease. This issue is especially relevant to CaOx nephrolithiasis, in which tubular crystal–epithelial interactions and papillary/RP-associated mineralization arise in distinct cellular environments. A candidate target must therefore combine causal relevance to stone-associated pathology with sufficient molecular and spatial selectivity to permit intervention without disrupting its physiological functions in other tissues [115].

The experimental models used to establish these mechanisms impose another limitation. COM-exposed tubular cells and rodent models of hyperoxaluria or glyoxylate-induced CaOx deposition are well suited to studying epithelial injury and renal crystal deposition, but neither fully reproduces the prolonged processes underlying mature human stone formation and recurrence. RP studies bring the evidence closer to human stone pathology, yet therapeutic intervention in this setting remains largely unexplored [70,71,72]. Improvement in oxidative injury, cell death, or renal crystal burden should therefore not be assumed to indicate prevention of clinically relevant stone formation. Moving beyond experimental proof of concept will also require adequate renal exposure, a workable dosing window, and long-term safety.

Translational development should initially focus on CaOx-validated ubiquitin-regulatory nodes for which causal manipulation produces reproducible disease-relevant phenotypes and selective pharmacological modulation appears feasible. Candidates meeting these criteria can then be evaluated using kidney- or cell-selective delivery and in models that better capture recurrent or plaque-associated stone formation [110,116]. Progress toward clinical application will ultimately require confirmation of target relevance in human disease tissue, identification of the patients most likely to benefit, and evidence that pathway modulation improves stone-relevant outcomes without compromising physiological proteostasis.

## 7. Conclusions and Future Directions

Ubiquitin-dependent protein regulation is emerging as an important component of CaOx nephrolithiasis. Disease-specific studies now link ubiquitin-regulatory mechanisms to tubular injury, ferroptosis, crystal deposition, and RP-associated papillary mineralization [16,55,70,71,72,85,86,102]. Together, these findings suggest that altered ubiquitin-dependent protein regulation may influence both crystal–epithelial responses and the persistence of a stone-promoting renal environment. The evidence remains uneven, however, and the mechanisms identified to date likely represent only a small fraction of the ubiquitin-regulatory network involved in stone formation.

A central unresolved question is whether these individual mechanisms reflect coordinated remodeling of the ubiquitin system in CaOx disease. Most studies have focused on single E3/DUB–substrate relationships, while direct CaOx-specific mechanisms remain scarce and many plausible pathways are derived from other renal or non-renal settings. It remains unclear whether CaOx exposure elicits a characteristic ubiquitin signature, which components are specific to stone-associated pathology rather than generic renal injury, and how these responses vary across renal cell types and anatomical compartments.

Resolving these questions will require more systematic and spatially resolved analysis. Establishing CaOx specificity will require direct comparison with other forms of renal injury to distinguish shared stress-responsive ubiquitin changes from alterations preferentially associated with crystal exposure. Disease-enriched ubiquitin regulation should further be resolved at the substrate, cell-type, and renal-compartment levels and linked to stone-relevant phenotypes such as crystal adhesion, retention, deposition, or RP-associated mineralization. Quantitative ubiquitin-remnant and chain-resolved profiling integrated with proteomics and phosphoproteomics could define disease-associated changes in ubiquitin regulation [117], while single-cell and spatial approaches could localize candidate pathways to crystal-exposed tubular segments or papillary/RP-associated compartments [118]. Candidate mechanisms will then require causal validation in kidney-relevant models and assessment of their conservation in human stone-associated tissues. Defining a reproducible CaOx-specific ubiquitin landscape would help distinguish adaptive responses from changes that sustain tissue injury, crystal retention, or papillary mineralization and identify disease-relevant ubiquitin-regulatory nodes for subsequent therapeutic investigation.

## Figures and Tables

**Table 1:** Direct and supportive ubiquitin-related evidence in calcium oxalate nephrolithiasis and Randall’s plaque.

Regulator/Pathway	Target	Ubiquitin-Related Mechanism	Experimental Context	CaOx/RP Relevance	Evidence Level	Ref.
VHL	BICD2	Promotes K48-linked polyubiquitination and proteasomal degradation of BICD2	CaOx-exposed renal epithelial cells; Vhl-mutant mice	Ferroptosis and renal injury	Level I	[102]
STUB1	CFTR	Promotes CFTR ubiquitination and degradation	COM-treated HK-2 cells; glyoxylate-treated mice	Tubular injury and crystal deposition	Level I	[16]
Lgals3-TRIM21	PKM2	Interferes with TRIM21-mediated PKM2 ubiquitination and degradation	COM-treated HK-2 cells; CaOx mice	Crystal deposition and renal fibrosis	Level I	[85]
PRMT1-UBE2M-NEDD4	PPARγ	Promotes NEDD4-dependent PPARγ ubiquitination and degradation through UBE2M methylation and NEDD4 neddylation	COM-treated tubular cells; tubular-specific Prmt1 mice	Metabolic dysfunction and renal injury	Level I	[86]
Hsp90α-associated regulation	GPX4	Promotes GPX4 ubiquitination and degradation; responsible E3 undefined	NaOx-treated HK-2 cells; high-oxalate-diet mice; tubular Hsp90aa1 cKO mice	Ferroptosis, crystal adhesion and deposition	Level I	[55]
SIRT6 ubiquitination/HSP70	SIRT6	SIRT6 ubiquitination followed predominantly by autophagy–lysosomal degradation	Hyperoxaluria/CaOx models; human stone-associated samples	Tubular DNA damage and crystal nephropathy	Level I	[87]
OLMALINC	OCT4	Suppresses OCT4 ubiquitination and increases protein stability	Human RP tissue; osteogenic hRIFs	Osteogenic-like differentiation and RP formation	Level I	[70]
OMD-NEDD4	BMP2	Inhibits NEDD4-mediated BMP2 ubiquitination and proteasomal degradation	Human RP tissue; hRIFs	Osteogenic remodeling and RP formation	Level I	[71]
TRAF1-TRAF2/CBLC	TRAF1/TRAF2	Limits CBLC-mediated ubiquitination and degradation of TRAF1/TRAF2	Human RP tissue; AQP2+ collecting duct cells; RP models	Collecting-duct apoptosis, calcium deposition and RP formation	Level I	[72]
CAV1-associated proteostasis	CAV1	Proteasome-dependent CAV1 loss; direct ubiquitination not demonstrated	COM-treated HK-2 cells; CaOx mice	ER stress and tubular injury	Level II	[75]
Macrophage-derived TGF-β1/UPS signaling	RhoA	Proteasome-dependent RhoA loss; direct ubiquitination not demonstrated	CaOx macrophage-conditioned medium; MDCK cells	Tubular EMT and fibrosis	Level II	[78]
Crystal-induced proteostasis alteration	Global ubiquitinated proteins	Reduces global ubiquitinated proteins; no specific E3/DUB-substrate axis identified	COM/COD-exposed renal tubular cells	Oxidative stress, cell death and crystal adhesion	Level II	[88]

Abbreviations: CaOx, calcium oxalate; COD, calcium oxalate dihydrate; COM, calcium oxalate monohydrate; DUB, deubiquitinating enzyme; EMT, epithelial–mesenchymal transition; GPX4, glutathione peroxidase 4; hRIF, human renal interstitial fibroblast; RP, Randall’s plaque; UPS, ubiquitin-proteasome system. Evidence levels: Level I, direct CaOx/RP-specific evidence for ubiquitin-dependent regulation; Level II, supportive CaOx-context evidence implicating UPS/proteostasis without a fully established substrate-specific ubiquitination mechanism.

**Table 2:** Representative ubiquitin-dependent mechanisms from other disease contexts relevant to calcium oxalate nephrolithiasis.

CaOx-Relevant Pathological Process	Regulator-Target	Ubiquitin-Related Mechanism	Experimental Context	Ubiquitin Linkage/Site	Ref.
NF-κB inflammatory signaling	A20-RIP1/TRAF6	Edits ubiquitin chains on RIP1/TRAF6 to restrain NF-κB signaling	TNF/TLR-stimulated immune-cell models	K63 deubiquitination; K48-linked RIP1 ubiquitination	[80]
Oxidative injury and ferroptosis	TRIM21-GPX4	Promotes GPX4 ubiquitination and proteasomal degradation	Renal I/R mice; HK-2 H/R cells	Not defined	[103]
Oxidative injury and ferroptosis	OTUD5-GPX4	Stabilizes GPX4; OTUD5 loss promotes its ubiquitination and degradation	Tubular-specific Otud5 knockout mice; renal I/R and tubular H/R models	Not defined	[104]
Inflammasome activation	TRIM31-NLRP3	Promotes NLRP3 polyubiquitination and proteasomal degradation	Macrophages; inflammatory mouse models	K48-linked; site undefined	[83]
Inflammasome activation	BRCC3-NLRP3	Deubiquitinates NLRP3 and promotes inflammasome activation	Macrophage/immune-cell models	Not defined	[82]
Epithelial surface remodeling and crystal adhesion	USP10-CD44	Deubiquitinates and stabilizes CD44	Breast cancer cells and mouse tumor models	Not defined	[76]
HA-rich epithelial surface and crystal interaction	USP17/USP4-HAS2	USP17 removes HAS2 polyubiquitin chains; USP4 reduces monoubiquitination	HEK293T and cancer-related cell models	K48/K63 poly-Ub (USP17); HAS2 K190 mono-Ub	[77]

Abbreviations: CaOx, calcium oxalate; GPX4, glutathione peroxidase 4; H/R, hypoxia/reoxygenation; HA, hyaluronan; HAS2, hyaluronan synthase 2; I/R, ischemia/reperfusion; NF-κB, nuclear factor κB; NLRP3, NOD-like receptor family pyrin domain-containing 3; RIP1, receptor-interacting protein 1; TLR, Toll-like receptor; TNF, tumor necrosis factor.

## Data Availability

No new data were created or analyzed in this study. Data sharing is not applicable to this article.

## Supplementary Materials

The following supporting information can be downloaded at: https://www.mdpi.com/article/10.3390/biomedicines14102316/s1, Supplementary Figure S1. Structured search and screening flow for CaOx/Randall’s plaque-specific ubiquitin evidence. Supplementary Table S1. Literature-search strategies for CaOx/Randall’s plaque-specific and contextual ubiquitin evidence.
