# Supplementary material for: From Proteostasis Disruption to Stone Formation: Ubiquitin-Dependent Regulatory Networks in Calcium Oxalate Nephrolithiasis
Source: Biomedicines. 2026 Oct 12;14(10):2316. doi: 10.3390/biomedicines14102316 (PMC13701867; doi:10.3390/biomedicines14102316)
Supplement: Supplementary file 1 [file biomedicines-14-02316-s001.zip › Supplementary Table S1.pdf]

Supplementary Table S1. Literature-search strategies for CaOx/Randall's plaque-specific and contextual ubiquitin evidence

Part A. Structured disease-specific search for CaOx/Randall's plaque ubiquitin evidence

Search date: September 24, 2026. Coverage: database inception to September 24, 2026. Restrictions: none for language, publication year, or document type.

Source	Search field	Records retrieved	Notes
PubMed/MEDLINE	Title/Abstract	40	Electronic database search
Web of Science Core Collection	Topic (TS)	39	Electronic database search
Scopus	Title, Abstract, Keywords	196	Electronic database search
Reference-list screening	Reference lists of eligible/key mechanistic studies	1	Additional original study

PubMed/MEDLINE — Title/Abstract

```
(
"calcium oxalate"[Title/Abstract]
OR "calcium oxalate monohydrate"[Title/Abstract]
OR CaOx[Title/Abstract]
OR nephrolithiasis[Title/Abstract]
OR urolithiasis[Title/Abstract]
OR "kidney stone"[Title/Abstract]
OR "kidney stones"[Title/Abstract]
OR "renal stone"[Title/Abstract]
OR "renal stones"[Title/Abstract]
OR "urinary stone"[Title/Abstract]
OR "urinary stones"[Title/Abstract]
OR "Randall's plaque"[Title/Abstract]
OR "Randall plaque"[Title/Abstract]
OR "Randall's plaques"[Title/Abstract]
OR "Randall plaques"[Title/Abstract]
OR "renal papilla"[Title/Abstract]
OR "renal papillae"[Title/Abstract]
OR "papillary calcification"[Title/Abstract]
OR nephrocalcinosis[Title/Abstract]
OR "renal calcification"[Title/Abstract]
OR "crystal deposition"[Title/Abstract]
OR "oxalate nephropathy"[Title/Abstract]
OR "oxalate crystal"[Title/Abstract]
OR "oxalate crystals"[Title/Abstract]
)
AND
(
ubiquitin*[Title/Abstract]
OR ubiquitinat*[Title/Abstract]
OR ubiquitylat*[Title/Abstract]
OR "ubiquitin-proteasome"[Title/Abstract]
OR "ubiquitin proteasome"[Title/Abstract]
OR "ubiquitin-proteasome system"[Title/Abstract]
OR "ubiquitin proteasome system"[Title/Abstract]
OR "E3 ligase"[Title/Abstract]
OR "E3 ligases"[Title/Abstract]
OR "ubiquitin ligase"[Title/Abstract]
OR "ubiquitin ligases"[Title/Abstract]
OR deubiquitin*[Title/Abstract]
OR deubiquitylat*[Title/Abstract]
OR deubiquitinat*[Title/Abstract]
OR "ubiquitin-specific protease"[Title/Abstract]
OR "ubiquitin-specific proteases"[Title/Abstract]
OR proteasom*[Title/Abstract]
OR "proteasomal degradation"[Title/Abstract]
OR "proteasome-dependent degradation"[Title/Abstract]
OR "protein degradation"[Title/Abstract]
OR "protein stability"[Title/Abstract]
OR neddylat*[Title/Abstract]
OR NEDD8[Title/Abstract]
OR "ubiquitin-like modification"[Title/Abstract]
OR "ubiquitin-like modifications"[Title/Abstract]
)
```

Web of Science Core Collection — Topic (TS)

```
TS=(
(
"calcium oxalate"
OR "calcium oxalate monohydrate"
OR CaOx
OR nephrolithiasis
OR urolithiasis
OR "kidney stone*"
OR "renal stone*"
OR "urinary stone*"
```

```
OR "Randall's plaque*"
OR "Randall plaque*"
OR "renal papilla*"
OR "papillary calcification"
OR nephrocalcinosis
OR "renal calcification"
OR "crystal deposition"
OR "oxalate nephropathy"
OR "oxalate crystal*"
)
AND
(
ubiquitin*
OR ubiquitinat*
OR ubiquitylat*
OR "ubiquitin-proteasome"
OR "ubiquitin proteasome"
OR "ubiquitin-proteasome system"
OR "ubiquitin proteasome system"
OR "E3 ligase*"
OR "ubiquitin ligase*"
OR deubiquitin*
OR deubiquitylat*
OR deubiquitinat*
OR "ubiquitin-specific protease*"
OR proteasom*
OR "proteasomal degradation"
OR "proteasome-dependent degradation"
OR "protein degradation"
OR "protein stability"
OR neddylat*
OR NEDD8
OR "ubiquitin-like modification*"
)
)
```

Scopus — Title, Abstract, Keywords

```
TITLE-ABS-KEY (
(
"calcium oxalate"
OR "calcium oxalate monohydrate"
OR CaOx
OR nephrolithiasis
OR urolithiasis
OR "kidney stone*"
OR "renal stone*"
OR "urinary stone*"
OR "Randall's plaque*"
OR "Randall plaque*"
OR "renal papilla*"
OR "papillary calcification"
OR nephrocalcinosis
OR "renal calcification"
OR "crystal deposition"
OR "oxalate nephropathy"
OR "oxalate crystal*"
)
AND
(
ubiquitin*
OR ubiquitinat*
OR ubiquitylat*
OR "ubiquitin-proteasome"
OR "ubiquitin proteasome"
OR "ubiquitin-proteasome system"
OR "ubiquitin proteasome system"
OR "E3 ligase*"
OR "ubiquitin ligase*"
OR deubiquitin*
OR deubiquitylat*
OR deubiquitinat*
OR "ubiquitin-specific protease*"
OR proteasom*
OR "proteasomal degradation"
OR "proteasome-dependent degradation"
OR "protein degradation"
OR "protein stability"
OR neddylat*
OR NEDD8
OR "ubiquitin-like modification*"
)
)
```

Reference-list screening

Reference lists of eligible articles and key mechanistic studies were screened to identify relevant original studies that might have been missed because ubiquitin-related mechanisms were not explicitly described in the title, abstract, or keywords. One additional eligible original study was identified.

Part B. Targeted supplementary searches for contextual ubiquitin mechanisms relevant to CaOx pathophysiology

Targeted supplementary searches were performed in PubMed/MEDLINE, Web of Science Core Collection, and Scopus through September 24, 2026 to identify representative original studies describing ubiquitin-dependent regulation of biological processes and molecular targets relevant to CaOx pathophysiology. These searches were mechanism-oriented and were used to support the contextual evidence summarized in Table 2; they were not designed as a second systematic or exhaustive disease-specific search.

CaOx-relevant process	Target concepts	Ubiquitin-related concepts	Representative targeted search structure	Purpose
Organelle quality control / tubular injury	PEX5; pexophagy; peroxisome	USP30; ubiquitin*; deubiquitin*; protein stability	(PEX5 OR pexophagy OR peroxisom*) AND (USP30 OR ubiquitin* OR deubiquitin* OR "protein stability")	Identify ubiquitin-dependent regulation of peroxisomal quality control relevant to tubular injury.
Oxidative injury / ferroptosis	GPX4; ferroptosis	TRIM21; OTUD5; ubiquitin*; E3 ligase*; deubiquitin*; proteasomal degradation	(GPX4 OR ferroptosis) AND (TRIM21 OR OTUD5 OR ubiquitin* OR "E3 ligase*" OR deubiquitin* OR "proteasomal degradation")	Identify regulator-substrate mechanisms controlling GPX4 stability and ferroptotic injury.
Tubular stress / inflammatory signaling	DUSP10; MAPK; JNK; p38; NF-kB	TRIM29; ubiquitin*; E3 ligase*; proteasomal degradation	(DUSP10 OR MAPK OR JNK OR p38 OR "NF-kB") AND (TRIM29 OR ubiquitin* OR "E3 ligase*" OR "proteasomal degradation")	Identify renal ubiquitin-dependent mechanisms linking tubular stress to inflammatory signaling.
Inflammasome activation	NLRP3; inflammasome	TRIM31; BRCC3; ubiquitin*; deubiquitin*; E3 ligase*	(NLRP3 OR inflammasome) AND (TRIM31 OR BRCC3 OR ubiquitin* OR deubiquitin* OR "E3 ligase*")	Identify E3- and DUB-mediated regulation of inflammasome activation.
Crystal-interacting epithelial surface	CD44; HAS2; hyaluronan	USP10; USP17; USP4; ubiquitin*; deubiquitin*; protein stability	(CD44 OR HAS2 OR hyaluronan) AND (USP10 OR USP17 OR USP4 OR ubiquitin* OR deubiquitin* OR "protein stability")	Identify ubiquitin-dependent control of epithelial-surface molecules potentially relevant to crystal interaction/adhesion.
