# Supplementary material for: From Proteostasis Disruption to Stone Formation: Ubiquitin-Dependent Regulatory Networks in Calcium Oxalate Nephrolithiasis
Source: Biomedicines. 2026 Oct 12;14(10):2316. doi: 10.3390/biomedicines14102316 (PMC13701867; doi:10.3390/biomedicines14102316)
Supplement: Supplementary file 1 [file biomedicines-14-02316-s001.zip › Supplementary Figure S1.pdf]

Supplementary Figure S1. Structured search and screening flow for CaOx/Randall’s plaque-specific ubiquitin evidence

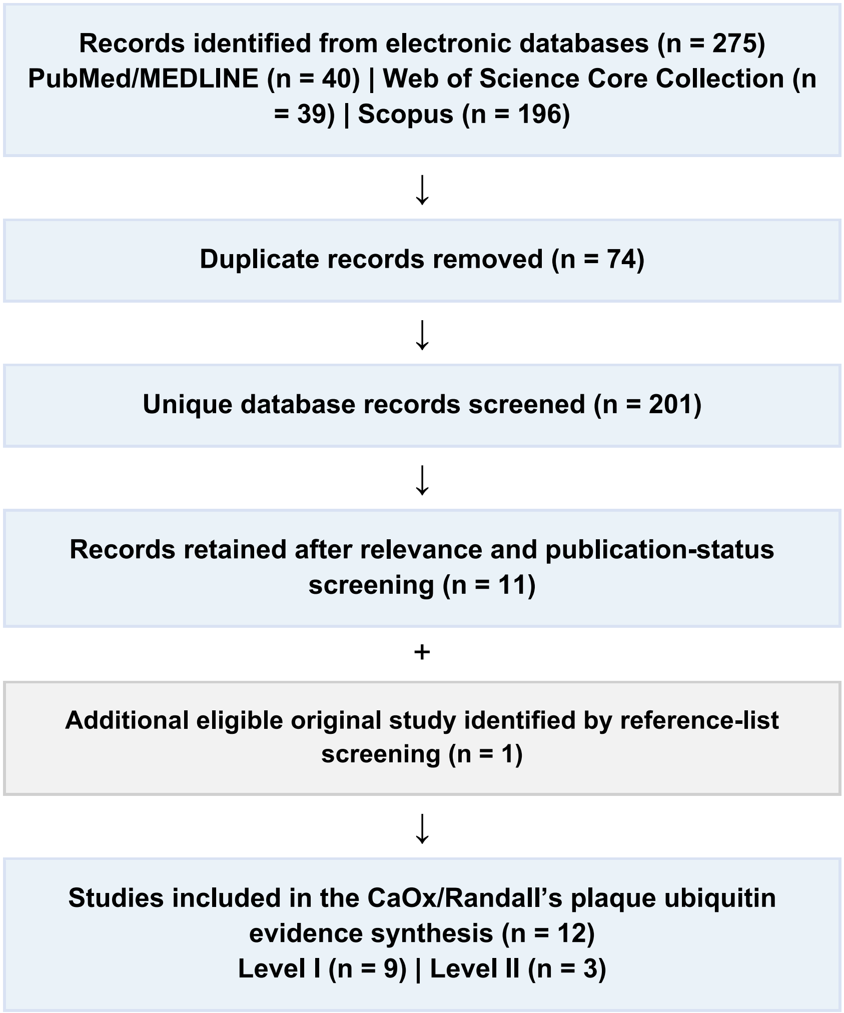

Supplementary Figure S1. Flow of the structured literature search used to identify direct and supportive ubiquitin-related evidence in calcium oxalate (CaOx) nephrolithiasis and Randall’s plaque (RP). Electronic searches were conducted in PubMed/MEDLINE, Web of Science Core Collection, and Scopus from database inception through September 24, 2026. After deduplication, records were screened for mechanistic relevance and publication status. One additional eligible original study was identified by reference-list screening. Retracted publications were excluded from the evidence synthesis. Level I denotes direct CaOx/RP-specific ubiquitin evidence; Level II denotes supportive CaOx-context evidence without a complete disease-specific ubiquitin causal chain.
